# Individual and Combined In Vitro Effects of Deoxynivalenol and Zearalenone on Boar Semen

**DOI:** 10.3390/toxins12080495

**Published:** 2020-08-01

**Authors:** Panagiotis D. Tassis, Ioannis A. Tsakmakidis, Veronika Nagl, Nicole Reisinger, Eleni Tzika, Christiane Gruber-Dorninger, Ilias Michos, Nikolaos Mittas, Athina Basioura, Dian Schatzmayr

**Affiliations:** 1 Farm Animals Clinic, School of Veterinary Medicine, Aristotle University of Thessaloniki, 54627 Thessaloniki, Greece; iat@vet.auth.gr (I.A.T.); eltzika@vet.auth.gr (E.T.); imichos84@hotmail.com (I.M.); basioura@vet.auth.gr (A.B.); 2 BIOMIN Research Center, Technopark 1, 3430 Tulln, Austria; veronika.nagl@biomin.net (V.N.); nicole.reisinger@biomin.net (N.R.); christiane.gruber@biomin.net (C.G.-D.); dian.schatzmayr@biomin.net (D.S.); 3 Department of Chemistry, School of Science, International Hellenic University, 65404 Kavala, Greece; nmittas@chem.ihu.gr

**Keywords:** deoxynivalenol, zearalenone, boar, mycotoxins, semen, reproduction, motility, spermatozoa, fertility, combined effects

## Abstract

Mycotoxins deoxynivalenol (DON) and zearalenone (ZEN) can negatively affect pig health. However, little is known about their effects on boar semen. We assessed the individual and combined effects of DON and ZEN on boar semen in vitro. In a pretrial, we determined the minimum dose (MiD) of each mycotoxin that induces a significant alteration of sperm progressive motility, as investigated using computer-assisted semen analysis (CASA). In the main trial, the individual and combined effects of each mycotoxin’s MiD on sperm motility and kinetics (CASA analysis), morphology (SpermBlue staining), viability (calcein-propidium iodide staining), membrane functional status (hypoosmotic swelling test), and chromatin integrity (acridine orange staining) were analyzed. Pretrial results suggested a MiD of 50.6 μM and 62.8 μM for DON and ZEN, respectively. In the main trial, DON and ZEN administered at MiD significantly affected CASA parameters (e.g., increase of immotile spermatozoa, reduction of progressive motile spermatozoa), decreased sperm viability, and affected sperm morphology (head abnormalities) and membrane functional status. DON and ZEN showed less than additive effects on most parameters tested and a synergistic effect on viability and on two CASA parameters. In conclusion, DON and ZEN showed individual and combined toxic effects on boar semen in vitro.

## 1. Introduction

Research efforts over half a century have proven that mycotoxins can pose a significant threat to health and reproductive efficiency of swine. They are secondary metabolites of certain fungi (genera *Aspergillus*, *Penicillium*, *Fusarium*, *Alternaria*, and *Claviceps*) that can be found in grains (e.g., maize, wheat, barley) worldwide [1]. They are produced before harvest of grains (fungi as plant pathogens), or during storage (fungi growing saprophytically). Data at a global scale showed that up to 80% of feed and food crops are contaminated with mycotoxins [2,3,4], and that co-contamination of grains with more than one mycotoxin is common [3,5]. Mycotoxin interactions frequently result in synergistic or additive effects, while in some studies antagonism has also been reported [5]. Pigs are exposed to mycotoxins through ingestion of contaminated feed, and *Fusarium* mycotoxins seem to be particularly significant due to their harmful effects on swine health and performance [6]. Zearalenone (ZEN) often co-occurs with deoxynivalenol (DON) in grains with varying contamination levels depending on factors such as region, country or climate [7]. Presentation of analysis from samples from more than 100 countries suggested that DON and ZEN are among the top three mycotoxins in complete animal feed and feedstuffs, with occurrence in 64% and 45% of all samples, respectively, and 388 μg/kg and 55 μg/kg median concentration among the positive samples, respectively [3]. According to the European Commission [8], the maximum recommended contamination levels in swine feed are 0.9 mg DON/kg feed, 0.1 mg ZEN/kg feed for piglets and gilts and 0.25 mg ZEN/kg feed for sows and fattening pigs, respectively.

ZEN is a phenolic resorcyclic acid lactone mycotoxin produced by Fusarium species, in grains. ZEN acts by binding estrogen receptors (ERs), with a stronger affinity to ER-α compared to ER-β. Briefly, ZEN metabolic pathway after consumption of contaminated feed includes rapid absorption from the intestine and extensive liver phase I and II biotransformations [9]. Nevertheless, gastro-intestinal microbial activity (pre-absorptive ZEN modification) that leads to the detection mainly of α-zearalenol has been demonstrated with the use of porcine chyme (collected from caecum, colon, and rectum) in vitro [10]. The intestinal mucosa and the liver play a critical role in ZEN metabolic pathways, whereas hepatic and ovarian 3α- and 3β-hydroxysteroid dehydrogenase can reduce ZEN. Phase I metabolism results in α-zearalenol (α-ZEL) and β-zearalenol (β-ΖΕL), both the main reductive metabolites of ZEN, as well as α- and β-zearalanol and zearalanone. The α-zearalenol is the main form observed in swine, that presents estrogenic potency greater than the parental toxin. Phase II includes glucuronidation of ZEN and phase I reduced forms (catalyzed by uridine diphosphate glucuronyl transferase), towards polar forms which lack estrogenic potency. Glucuronides further undergo biliary or urinary excretion. Significant re-absorption in the intestinal tract influences ZEN excretion via enterohepatic circulation [9,11]. ZEN absorption reaches 61–85%, but only 1.8% of the parent toxin is systemically available due to extensive first pass metabolism in intestine and liver. Oral administration of 1 mg ZEN/kg body weight resulted in a maximum blood serum concentration of 3.6 nΜ ZEN (range 0.89–5.5 nM) [12].

DON complex metabolic pathway includes conversion of the toxin to degradation derivatives mediated by microbes in the digestive tract, as well as the involvement of intestinal mucosa, liver, and kidneys in the phase II metabolism. De-epoxy-DON (DOM-1) is the de-epoxidized form that is produced through microbial metabolism of the parent toxin, whereas phase II metabolism results in conjugated forms of DON and DOM-1 with glucuronic acid that further facilitate excretion via urine. De-epoxidation degree is greater in the distal than the proximal part of the digestive system and reaches almost 100% in rectal feces. However, DON is almost completely absorbed in the upper digestive tract, thus partially “avoiding” microbial de-epoxidation [13]. An entero-hepatic cycling of both DON and DOM-1 has been already suggested [14], whereas the ability of pigs to de-epoxidize DON is probably acquired and could be age-related [15]. Previous in vitro studies regarding the effects of DOM-1 and DON-glucuronide on the viability of various cell lines, have suggested them as either potentially less toxic (DOM-1) or not toxic (DON-glucuronide) in comparison with DON [16,17]. DON systemic bioavailability is the highest in pigs and ranges between 52.7% and 100% after oral exposure [18]. In a previous study [19] with growing pigs that received 5.7 mg DON/kg diet for 4 or 6 weeks, maximum serum levels of 17.1–26.3 ng free DON/mL serum where observed and a greater bioavailability was determined in animals fed chronically in comparison with pigs fed the same diet as one meal (i.e., acute exposure). After intravenous application of DON in pigs (40 days of age) blood plasma mean concentration of 9.22 ng DON/mL (administration of 250 μg/kg body weight) or 26.8 ng DON/mL (administration of 750 μg/kg body weight) was reported [20].

The significance of boar semen characteristics in the reproductive success and improvement of traits of economic importance (i.e., growth rate, feed conversion efficiency) in farrow-to-finish farms is undisputable [21]. Intensive reproductive performance with the use of hyperprolific sows requires utilization of high-quality boar semen that would support such reproduction pace [22]. Approximately 90% of swine reproduction has been performed with artificial insemination in the past two decades, 99% of which has been performed with the use of liquid semen and ambient temperature extenders [21,22].

Semen viability, motility, and other characteristics can be negatively affected by several factors such as bacteria (e.g., *Escherichia coli*), viruses (e.g., porcine reproductive and respiratory syndrome virus), and mycotoxins [21,23]. Even though the particular mechanisms of mycotoxin-induced negative effects on boars have not been fully explored, it has been indicated that mycotoxins, such as Fumonisin B1 (FB1) at contamination levels of 5, 10 or 15 mg/kg feed in pigs (6 months duration of feeding), or other mycotoxins (DON, ZEN, aflatoxin B1 (AFB1), T-2, ochratoxin A (OTA), patulin, citrinin) in trials with rabbits, horses, mice or rats can be associated with reduced vigor and reproductive performance in males, as reviewed by El Khoury and coworkers [24]. Aflatoxins, ergot alkaloids, fumonisins (FBs), and trichothecenes (e.g., DON, T2 toxin, OTA, and ZEN) are considered significant for their effects on boar health [25,26].

DON, ZEN, FB1, and OTA have been tested in vitro or in vivo for their toxic effects on semen of various species such as rabbits, rats, horses, and pigs [24,26,27,28], whereas due to the species-related increased sensitivity, ZEN has been investigated more intensively in swine [29]. The ability of DON to exert an adverse effect on the epididymis in a 90-day feeding trial with 10 ppm DON in mice, has been previously demonstrated [30], while also in a later study, an increase in germ cell degeneration, sperm retention, and abnormal nuclear morphology has been associated with ingestion of 2.5 mg DON/kg feed and 5.0 mg DON/kg feed for 28 days in rats [31].

In regard to the effects of mycotoxins on boar fertility and semen characteristics, ZEN has been associated with alterations such as reduced serum testosterone levels, libido (feeding 40 mg ZEN/kg feed between 14 and 18 weeks of age) [32], testis weights (30% reduced weight after ingestion of 0.5–0.6 mg ZEN/kg feed for 64 days) [33], and spermatogenesis (9 mg ZEN/kg feed from 32 d of age up to 145 or 312 d of age) [34]. In vitro studies have proven that ZEN and its major metabolites α- and β-zearalenol can decrease sperm viability and progressive motility (ZEN, α-ZEL, β-ZEL at 1 × 10^−8^ and 1 μM or 2 × 10^−7^ and 20 μM for 5, 16, 24 or 48 h [35]; ZEN and α-ZEL at 125, 187.5 or 250 μM [36]). Additionally, ZEN and α-zearalenol can reduce the ability of boar spermatozoa to bind to the zona pellucida (125.6, 188.4, and 251.2 μM ZEN, as well as 124.8, 187.2, and 249.6 μM α-zearalenol for 1, 2, 3 or 4 h) [37] and affect sperm chromatin integrity of boars (31.4, 62.8, and 94.2 μM ZEN, as well as α-zearalenol at levels of 31.2, 62.4, and 93.6 μM for 4 h) [38]. Thus, they can reduce fertilization ability and normal embryonic development.

Even though pigs are known to be highly sensitive to DON [39], and DON possibly affects reproductive performance of sows through impairment of oocytes developmental competence, disorders of oocyte maturation and formation of embryos with abnormal ploidy [40,41], there is an absence of studies investigating its effect on boar semen characteristics. Furthermore, although DON and ZEN often co-occur in swine feed and their interactions have been demonstrated in various tissues [11,42] no previous research has described the combined effects of those two mycotoxins on boar semen characteristics.

The aim of this study is to evaluate the effects of DON and ZEN on various boar semen characteristics (motility, morphology, viability, sperm membrane functionality, and chromatin integrity) in vitro. We first investigated the individual effects of DON and ZEN, and subsequently assessed their combined effects and determined the type of interaction (antagonistic, synergistic or additive).

## 2. Results

### 2.1. Pretrial Tests

First, a pretrial was conducted to determine potential negative effects of the used solvent (dimethyl sulfoxide, DMSO) on boar semen progressive motility, which was considered as the primary parameter. Results indicated that tested levels of semen diluted with 0.7–1.2% (*v*/*v*) DMSO did not induce significant alterations of progressive motility in comparison with non DMSO-treated semen samples (control), at hour 1 of evaluation. Results of comparisons between DMSO-treated and control samples were for 0.7%: *t*-test = −1.572, *p* = 0.118; 0.8%: *t*-test = −1.119, *p* = 0.265; 0.9%: *t*-test = −1.924, *p* = 0.057; and 1.2%: *t*-test = −1.944, *p* = 0.054 (Figure S1). The visual inspection of residual plots did not reveal any obvious departures from homoscedasticity. In addition, the normality assumptions were fulfilled. For safety reasons the lowest concentration of *v*/*v* 0.7% DMSO was selected for all mycotoxin concentrations in the second part of the pretrial and the main trial.

Next, the minimum dose (MiD) of DON and ZEN that could induce a significant effect on boar semen progressive motility at hour 1 of evaluation, was assessed. As regards to DON, analysis indicated significant main effects of both treatment (effect of DON) (F (5, 39.14) = 5.580, *p* = 0.001) and time (observation time point) (F (4, 220) = 26.683, *p* < 0.001) on the response variable. The pretrial computer-assisted semen analysis (CASA) evaluation of DON effects on progressive motility showed an effect of the toxin on the primary investigation parameter (*p* = 0.027 vs. DMSO group) from the level of 50.6 μM DON onwards. On the other hand, the interaction term treatment × time (F (20, 200) = 1.082, *p* = 0.371) did not present a significant term. The above-mentioned threshold concentration of 50.6 μM DON (MiD) was selected for further investigation in the main trial. Figure S2 presents alterations of progressive motility values of DON-treated boar semen, at the pretrial part of the study.

The results of ZEN pretrial tests indicated significant main effects of both treatment (effect of ZEN) (F (6, 28.84) = 22.373, *p* < 0.001) and time (F (4, 144) = 20.551, *p* < 0.001) on the primary parameter. Interestingly, the linear mixed effects (LME) model revealed a significant interaction between treatment and time (F (24, 144) = 1.795, *p* = 0.019). Based on that, we can infer that the effect of ZEN on progressive motility alterations depends also on the time of observation. Results showed that concentration level of 62.8 μM ZEN was the MiD that reduced the primary parameter in comparison with DMSO-treated semen samples at 1 h and 2 h. Nevertheless, after 3 and 4 h of incubation, an even lower level of 47.1 μM ZEN semen induced significant reduction in progressive motility. Therefore, the inclusion level of 62.8 μM ZEN semen (MiD) fulfilled selection criteria for further evaluation in the main trial. Figure S3 presents alterations of progressive motility mean values observed in pretrial tests after introduction of various ZEN concentrations in extended boar semen.

### 2.2. Main Trial

The aim of the main trial was to evaluate individual and combined effects of MiD of DON and ZEN on all CASA parameters, DNA integrity, hypoosmotic swelling test (HOST), viability, and morphological characteristics of boar semen. As previously mentioned, a control group of samples that received neither DMSO nor any of the tested mycotoxins was present in the trial design. Results of pretrial investigation proved absence of statistically significant differences between control semen without DMSO and 0.7% DMSO at all comparisons, groups, and time points. Therefore, results and comparisons between DMSO group and mycotoxin-treated groups were used for evaluation of effects and will be presented further in the main trial.

#### 2.2.1. CASA Results

The CASA evaluations demonstrated a significantly greater number of immotile and non-progressive motile spermatozoa in all mycotoxin-treated groups, either separately or simultaneously exposed (i.e., DON, ZEN, or DON + ZEN) irrespective of incubation time. Mean values of progressive motile spermatozoa at the DMSO vs. DON group comparison showed a trend towards significant decrease in the mycotoxin-treated group (*p* = 0.056). Similarly, the factor time significantly affected progressive motile spermatozoa irrespective to treatment, thus in all groups, especially in the first two hours of evaluation. The incubation time effect was absent in the last two time points of evaluation.

Rapid moving spermatozoa results showed that the effect of mycotoxins was time-dependent. The ZEN and DON + ZEN group showed a significant reduction of rapid motility at hour 0, representing a possible acute toxic effect from the beginning of evaluation. DON showed a trend towards a significant negative effect on this specific parameter at the 4th hour (*p* = 0.063) of measurements.

Medium motile spermatozoa values were significantly reduced only in ZEN-treated groups (alone or in combination with DON). That negative effect of ZEN was present at 0 h, disappeared at 1 h, and reoccurred at the last three time points of observations. A significant negative effect of DON alone was not present for this specific parameter.

Slow motile parameter results indicated a trend towards significant increase in DON group only at the 1st hour of measurements (*p* = 0.062). On the contrary, ZEN either alone or in combination with DON significantly increased slow motile spermatozoa when compared with DMSO group (all time points), and DON group (0–3 h).

The curvilinear velocity (VCL) values showed a tendency of DON to negatively affect the parameter (*p* = 0.098) after 2 h of incubation, but insignificant differences were observed in the subsequent time points. On the contrary, significantly lower VCL values were present in groups treated either with ZEN alone or in combination with DON, when compared with DMSO group at all time points. There was a significant difference between the ZEN and ZEN + DON group only at 0 h, with the combined mycotoxins group showing a greater VCL reduction than the ZEN group.

The VSL parameter alterations during the study illustrate the negative effect of ZEN either alone or in combination with DON. DON alone did not significantly affect this specific parameter. Similarly, average path velocity (VAP) alterations imply a negative effect of ZEN either alone or in combination with DON. DON showed a trend towards a significant negative effect on the specific parameter (*p* = 0.089). An independent time effect on this parameter was present during evaluations, except for the third hour of observation.

The parameters linearity (LIN), straightness (STR), wobble (WOB), and beat/cross frequency (BCF) were negatively affected in the ZEN and DON + ZEN groups in comparison with the DMSO group, while DON alone did not cause any significant alteration. The amplitude of lateral head displacement (ALH) parameter results showed a quite different pattern, since ZEN group values showed a trend towards significant difference when compared with the DMSO group, only at hour 0 (*p* = 0.074). On the other hand, combined exposure of boar semen to DON and ZEN resulted in significant reduction of ALH values at 0 h and after 2 h of incubation. At 1, 3, and 4 h of incubation insignificant ALH value differences were observed.

The parameter hyperactive spermatozoa was affected variably by mycotoxin exposure at 0 h, 1 h, and 2 h, while for the other time points (3 h and 4 h) no effect was present when compared with the DMSO group. At 0 h and 2 h, ZEN and DON + ZEN exposure resulted in significantly lower values in comparison with the DMSO group, while DON induced significant alterations only at 2 h. Moreover, at 1 h, a significant reduction of values was present only in the ZEN group. CASA parameters are presented in Table 1.

#### 2.2.2. Results on Morphology, Viability, HOST, and Nuclear Integrity

Alterations of spermatozoa head abnormalities during this study implied statistically significant main effects of both treatment (*p* < 0.001) and time (*p* < 0.001) on the examined parameter after dropping out the insignificant interaction term of treatment × time. The post-hoc analysis for the main effect of time indicated statistically significant differences between all pairwise comparisons up to the third hour of the study. Regarding the main effect of treatment, the analysis indicated statistically significant differences between ZEN either alone or in combination with DON compared to DMSO group. On the contrary, the findings did not reveal a statistically significant difference between DON and DMSO groups. The independent effect of time to all groups was significant up to the third hour of the study and then decreased to insignificant levels. The changes of semen quality characteristics are presented in Table 2, whereas a characteristic picture of observed morphological abnormalities during the study is demonstrated in Figure S4.

Alterations in viable spermatozoa after mycotoxin exposure, demonstrate a negative effect of ZEN exposure either alone or in combination with DON. However, DON also reduced values of live spermatozoa at a significant level at the fourth hour of observations. It should be stated that values between ZEN and DON + ZEN groups were significantly different at all time points, showing lower values in the combined exposure group. Figure S5 represents differences observed among viable and non-viable spermatozoa under the microscope (ZEN group, 4 h).

Results of sperm membrane functional status (HOST) test demonstrated statistically significant main effects of both treatment (*p* < 0.001) and time (*p* < 0.001) on the examined parameter after dropping out the insignificant interaction term of treatment × time. The post-hoc analysis for the main effect of time indicated statistically significant differences between all pairwise comparisons up to the fourth hour of the study. Regarding the main effect of treatment, the analysis indicated statistically significant differences between ZEN either alone or in combination with DON compared to DMSO group. Quite similarly a statistically significant difference between DON and DMSO groups was observed. Figure S6 demonstrates typical alterations observed in a ZEN group replicate (4 h). Regarding sperm nuclear chromatin integrity, almost all results of all groups and time points proved the absence of significant DNA damage. All DNA integrity impairment findings belonged to DON + ZEN-treated boar semen group (Figure S7). Figure S8 from the DMSO group (0 h) demonstrates normal spermatozoa with compact chromatin structure which fluoresce green. Three out of a total of 100 evaluations had a marginal shift from normality, one with 1% damage at 4 h, another with a similar damage at 0 h, as well as a third with 2% damage at 4 h. DNA integrity results did not undergo statistical evaluation.

#### 2.2.3. Combined Effects of DON and ZEN

All evaluations were carried out in comparison with DMSO group. Where treatment × time interaction was present, estimation of effects is presented at each individual time point, while in parameters where such interaction was absent, the total mean values were evaluated. Based on the evaluation of statistical differences, it became obvious that a less than additive effect of the two mycotoxins were present in most parameters. However, for particular parameters, such as rapid spermatozoa, VCL, and viability, synergistic interaction is evident. An antagonistic effect was not observed in any of the tested parameters. (Table 3).

## 3. Discussion

Based on the results of this study, DON and ZEN either alone or in combination can be considered as harmful to boar semen characteristics in vitro. Exposure to the trichothecene DON alone negatively affected two important CASA parameters (i.e., immotile and progressive motile spermatozoa), along with sperm morphology and viability, suggesting a toxic effect on boar semen. To our knowledge, no previous reports on effects of DON on boar semen in vitro are available.

However, studies on the effects of DON on semen of other species have showed interesting results. In a recent study on mice sperm, Salahipour and coworkers observed a dose-dependent toxic effect of 2.5–10 μM DON with a reduction in sperm metabolic activity and membrane integrity, and an increase in lipid peroxidation rate and DNA damage [44]. By contrast, in our study, toxic effects of DON on boar semen were not observed at concentrations up to 50.6 μM, and we did not detect an effect on DNA integrity. One explanation for these different outcomes may be differences in the experimental setup, since Salahipour and coworkers used a longer incubation time of 6 h. An alternative explanation could be that mouse semen is more sensitive to DON than boar semen. Indeed, DNA stability of spermatozoa has been shown to differ between species, according to a study that evaluated mammalian spermatozoa from eleven species [45]. It was shown that such differences may be due to differences in genomic structure, and the different ability of protamines (P1 and P2) to permit packaging of the DNA molecule, as well as differences in disulfide bonding, lysine, arginine, and cysteine residues in protamines. Spermatozoa of species lacking P2 resisted fragmentation more effectively in that study under the tested conditions (freeze/thawing and subsequent 4 h incubation at 5% CO_2_ in air at 37 °C), than those that contained both P1 and P2 [32]. In addition, it has been stated that even though boar semen has the gene for P2 protein, it seems that it is either dysfunctional, or produces an aberrant form of the protein [46].

A negative effect of ZEN was detected for most investigated parameters. Our findings extend previous knowledge in that field. A previous study demonstrated a negative effect of 125.6, 188.4, and 251.2 μM ZEN, as well as of 124.8, 187.2, and 249.6 μM α-zearalenol on the viability and motility of boar semen [37]. In this study we report that a lower concentration of ≥62.8 μM ZEN can induce significant detrimental effects on viability and motility of boar semen. Furthermore, an absence of ZEN-attributed negative effects on DNA integrity in our study is comparable with findings of a previous study where levels of 31.4, 62.8, and 94.2 μM ZEN, as well as levels of 31.2, 62.4, and 93.6 μM α-zearalenol affected DNA integrity only in one out of four boar semen samples after 4 h of incubation [38]. Therefore, an effect of ZEN on DNA integrity was concluded to be individual-dependent [38], which could also be related with the previously mentioned significant stability of boar semen [45]. In that previous study [38], neither ZEN, nor α-ZEL affected in vitro sperm motility. In contrast, in our study, which included a different number of samples and number of boars, similar concentrations of ZEN showed a negative effect of ZEN on boar semen motility parameters.

Observations of DON and ZEN interaction in the present study demonstrated a mixed picture that included less than additive effects in many parameters, as well as synergism in others. Thus, for certain parameters, when ZEN was applied in combination with DON, a significantly greater impact was detected compared to individual application of ZEN. For some of these parameters, DON did not show a significant effect when applied individually. Previous studies have evaluated combined effects of DON and ZEN in different tissues and species with varying results [47]. Synergism [5,48,49] or additive effects [5,50,51] of DON and ZEN have been previously demonstrated. In human colon carcinoma cells (HCT116), DON (100 μM) and ZEN (40 μM) caused a sub-additive effect on cell viability through the activation of the mitochondrial apoptotic process [52]. A previous study assessing combined effects of a 10 μM DON and 10 μM ZEN mixture on Caco-2 cells, suggested a far less than additive effect on the inhibition of DNA synthesis, whereas a synergistic effect was reported as regards to lipid peroxidation [53]. Inconsistent results among studies could be attributed to differences between species and cell types.

The design of this study also aimed at elucidating the mechanisms behind negative effects of DON and ZEN in boar semen. DON and ZEN were found to affect sperm membrane functionality. Alterations in membrane osmotic resistance, as the ability to control fluxes of electrolytes and non-electrolytes, as well as the damage of DNA structure of porcine sperm cells have been correlated with sperm motility and morphology, as well as with subsequent negative fertility results [54]. Significant negative effects of DON and ZEN detected in HOST support a key role of membrane functionality in motility, morphology, and viability of boar semen. Since integrity of the sperm outer membrane is essential for sperm metabolism, capacitation, oocyte binding, and acrosome reaction, the observed negative effect of both mycotoxins may have resulted in loss of homeostasis. Failure to restore homeostasis can trigger cell death, thus altering tested parameters of motility, morphology, and viability [52,55]. Apart from membrane osmotic resistance and DNA integrity, other possible targets of DON and ZEN toxicity in boar spermatozoa could be investigated in future studies. For example, DON and ZEN have been shown to cause mitochondrial damage in mouse Leydig tumor cells (MLTC-1) [56].

Motility and bacterial load are considered as the main quality parameters of preserved semen [57], whereas previous correlations between boar semen motility and other characteristics, and reproductive performance in vivo [36,58,59], suggested that factors capable of reducing boar semen motility could affect its fertilizing ability in vivo and consequently reproductive performance. Although progressive motility is one of the most important indicators of fertilizing ability in vivo [60], there are reports mentioning VSL, VAP, and ALH as significant parameters for the evaluation of boar semen used in artificial insemination [61,62]. All these parameters were significantly decreased after exposure to one or both mycotoxins in our study, thus supporting the hypothesis of a detrimental effect of DON and ZEN on fertility. Moreover, negative effects of DON and ZEN on HOST results support the aforementioned hypothesis, since membrane integrity is crucial to sperm survival inside the female reproductive tract [55,63] and osmotic resistance of porcine sperm cells is related to field fertility and litter size [63], as well as to in vitro fertility [64]. Based on the results of this study, it could be further discussed that co-exposure of boar semen to DON and ZEN could induce greater toxic effects on reproductive performance in vivo, than separate exposure. On the contrary, in two studies with very low mycotoxin levels by Sambuu and coworkers [65,66], absence of a negative effect of ZEN on boar semen fertilization capability was reported, either after fertilizing cumulus-oocyte complexes with spermatozoa in fertilization medium with 1–1000 μg ZEN/mL [65], or after culture of boar spermatozoa in semen storage medium containing up to 1000 μg ZEN/mL and 1000 μg α-ZEL/mL [66]. In contrast to previous evaluation with 40–80 times greater mycotoxin concentration levels [37], exposure to much lower levels (1000 μg ZEN/mL) during in vitro fertilization period had a positive effect on fertilization of oocytes [65]. After one week of exposure of boar semen to similar low doses of 1000 μg ZEN/mL and 1000 μg α-ZEL/mL, absence of differences in fertilization capability of oocytes was observed, as well as absence of significant negative effect on motility, viability, plasma membrane integrity, and acrosomal integrity of spermatozoa even after three weeks of storage [66]. Nevertheless, correlation of the DON or ZEN MiD levels with relevant DON or ZEN concentrations in feed that would induce such as an effect on boar semen in vivo is hard to establish. Even though metabolic properties of DON and ZEN and their metabolites, as regard to their transfer to boar seminal plasma have not been heavily investigated, MiD levels used in this study would probably correlate with extremely high concentration levels in feed in cases of acute exposure of boars. Further comparative in vitro and in vivo studies are needed to confirm such observations.

Considering that occurrence and co-occurrence of mycotoxins is common in feed [3,5], increased caution in minimizing the risk of mycotoxicosis is critical to boar health and spermatogenesis, and thus to proper reproductive performance. Therefore, prudent mycotoxin screening of boar diets is strongly recommended and actions against mycotoxins in feedstuffs should be taken on farms, including the disposal of contaminated grains and the application of mycotoxin deactivating feed additives.

## 4. Conclusions

In conclusion, concentrations ≥50.6 μM DON and ≥62.8 μM ZEN induced significant negative effects on boar semen characteristics in vitro suggesting a negative effect of these mycotoxins on boar fertility. When applied in combination, DON and ZEN exerted a greater effect on many tested parameters in vitro than each mycotoxin applied separately. Combined in vitro effects were in most cases less than additive and in some cases synergistic. Beyond the scope of the present study, probably extremely great DON and ZEN feed contamination levels would be needed to induce similar (to our in vitro results) in vivo observations at an acute case in boars.

## 5. Materials and Methods

The study was approved by the Research Committee of the Aristotle University of Thessaloniki, Greece (Code Nr: 92520, Scientific Responsible: P.D. Tassis). All examinations were performed in the Unit of Biotechnology of Reproduction of the Farm Animals Clinic, School of Veterinary Medicine, Aristotle University of Thessaloniki, Greece. The study comprised of two separate parts: a pretrial and a main trial.

### 5.1. Samples Origin and General Procedures

The semen samples were collected from active boars (13–14 months of age at the start of the study) Duroc x Pietrain hybrids which were vaccinated against major swine pathogens, while also preventive deworming was implemented at regular intervals. Boars were fed appropriately (corn/barley/soy mix) according to their nutritional requirements and water was offered ad libitum. The farm has its own feed mill to produce the boars’ feed. Mycotoxin analysis of feed was performed by LC/MS-MS according to regular farm schedule, four times per year. Briefly, for the detection of AFB1, DON, ZEN, OTA, and T-2 toxin the method described by Ren et al. [67] was followed, with sequential extraction of homogenized samples by 80% (*v*/*v*) acetonitrile aqueous solution, followed by filtration and purification with an appropriate MycoSep SPE column (Romer Labs, Tulln, Austria) and reconstitution in a suitable LC/MS-MS solvent for analysis. Fumonisins detection was performed with the method described by Li et al. [68] with extraction by 50% (*v*/*v*) of acetonitrile aqueous solution and collection of the supernatant for analysis. Results showed either absence (lower than detection limit) or traces of AFB1, DON, ZEN, OTA, FBs, and T-2 toxin below the maximum/guidance levels set by the European Commission [8,69]. Briefly 12 feed samples were tested, starting prior to the first semen sampling and ending at the last semen sampling. One feed sample was contaminated with 18.88 μg DON/kg and 1 μg T-2/kg feed, another sample had 17.65 μg DON/kg, 1.01 μg T-2/kg, and 3.39 μg OTA/kg feed, another had 12.29 μg DON/kg and 1 μg T-2/kg, whereas a fourth sample was contaminated with 33.92 μg DON/kg and 7.01 μg T-2/kg, and a fifth sample was contaminated with 7 μg AFB1/kg, 19.03 μg DON/kg, and 1.97 μg OTA/kg feed. All other samples had levels below detection limits for all tested mycotoxins. Boars were housed in separate pens next to the farm’s semen laboratory. Temperature and ventilation were automatically controlled in the boars’ room.

Each semen sample was prepared by two different boar ejaculates that were collected by the gloved hand method the same day and pooled. In total, 25 pooled boar semen samples that fulfilled the following quality criteria were included in the study (pretrial and main trial): viability >75%, total motility >60%, concentration >100 × 10^6^ spermatozoa/mL, morphological abnormalities <15%.

One-step semen dilution with a commercial extender (OPTIM-I.A^®^, Magapor, Spain) to a final concentration of 30 × 10^6^ spermatozoa/mL took place in the laboratory of the pig farm. Extended boar semen was transferred to the University’s laboratories with the use of portable refrigerating equipment (Klimabox, Minitube GmbH) within 60 min from the timepoint of collection. During transport, samples were kept at 16–18 °C.

Solid mycotoxin standards of DON and ZEN were purchased from Romer Labs (Tulln, Austria; >99% purity). All toxins were stored at −18 °C until use. For preparation of stock solutions regarding the MiD levels in the pre- and main trial, appropriate quantities of mycotoxins were dissolved in DMSO (D-4540, Sigma-Aldrich Corporation, St. Louis, MO, USA; >99.5% purity). Stock solutions for the individual toxin groups contained 7.23 µM DON and 8.97 µM ZEN, while for the combined toxin group the stock solutions contained 14.46 µM DON and 17.95 µM ZEN. By doing so, a final DMSO concentration of 0.7% was ensured for all treatment groups. In the same way stock solutions were prepared for testing the rest of the mycotoxin concentration in the pretrial (respective stock solutions molarities not presented). Thus, adequate amounts of toxin stock solutions were added to semen samples, and 3 mL aliquots (1 mL in the pretrial) were incubated in sterilized 10 mL tubes for 4 h (38.5 °C, 5% CO_2_, 100% humidity). At 0, 1, 2, 3, and 4 h of incubation, an appropriate semen volume was used to perform each analysis test as described in the following sections.

### 5.2. Pretrial

In the pretrial, we evaluated the effects of increasing DMSO concentrations on semen characteristics. Results of this pretrial were used to exclude potential negative effects of DMSO on investigated parameters in the main trial. To this end, semen samples (*n* = 5) were diluted with DMSO to final solvent concentrations (*v*/*v*) of 0.7%, 0.8%, 0.9%, 1.2%, 3%, and 5%, respectively. As a main parameter in this pretrial, sperm progressive motility was determined via computer-assisted semen analysis (CASA, details see below) after 1 h of incubation.

Afterwards, the minimum dose (MiD) of DON and ZEN, that significantly reduced progressive motility after 1 h of incubation, was determined. For those experiments, two control groups were implemented: undiluted semen (without DMSO, “Control”) and semen diluted with DMSO (*v*/*v*, 0.07%; “DMSO”). Only significant differences of mycotoxin-treated semen samples to both control groups were considered at this stage of the study for selection of appropriate mycotoxin levels for further investigation. For that, appropriate amounts of DON and ZEN stock solutions were added to semen samples (*n* = 5) to yield final toxin concentrations of 16.9, 33.8, 50.6, and 67.5 μM DON and 31.4, 47.1, 62.8, 94.2, and 125.6 μM ZEN.

### 5.3. Main Trial

The main trial followed the basic design of the pretrial. It was performed in ten replicates as suggested by appropriate sample size calculation. The effects of DON and ZEN MiD, administered either alone or in combination, were investigated on CASA parameters, sperm morphology, sperm viability, sperm membrane function, and chromatin integrity. Briefly, the following groups were created and tested.

- Control group (semen without addition of DMSO or mycotoxins)
- DMSO group (0.7% *v*/*v* DMSO)
- DON group (addition of 50.6 μM (MiD) DON)
- ZEN group (addition of 62.8 μM (MiD)ZEN)
- DON + ZEN group (addition of MiD DON + MiD ZEN)

Semen characteristics were assessed by the following methods:

(a) Sperm motility/kinetics (total motility, progressive motility, immotile, rapid, medium, slow spermatozoa, curvilinear velocity (VCL), straight line velocity (VSL), average path velocity (VAP), lateral head displacement (ALH), beat-cross frequency (BCF), hyper activation, straightness (STR), linearity (LIN), wobble (WOB)) were evaluated by CASA (Sperm Class Analyser^®^ v.5.2.0.0., Microptic S.L., Automatic Diagnostic Systems, Barcelona, Spain) and a microscope (×100; AXIO Scope A1, Zeiss, Jena, Germany) accomplished with a heating stage. A 10 μL semen sample was placed on the preheated Makler chamber (Makler^®^ counting chamber, 10 μm deep, Sefi Medical Instruments, Haifa, Israel) at 37 °C, and triplicates of the assessment were performed. CASA was configured as follows: 10 fields and >500 spermatozoa, 25 frames/s, region of particle control 10–18 microns, progressive movement of >45% of the indicator STR, circumferential movement <50% LIN, depth of field 10 microns, and temperature of the microscope plate at 37 °C. The number of objects incorrectly identified as spermatozoa was manually removed and final analysis was done for each sample.

(b) Sperm morphology was evaluated by the SpermBlue staining method (SpermBlue^®^ 08029, Microptic S.L., Barcelona, Spain) according to the manufacturer’s instructions. Spermatozoa were assessed microscopically (×400) and classified as normal or with morphological abnormalities (head, neck, mid place and tail, cytoplasmic droplets). One slide was prepared per sample, and 200 spermatozoa per slide were counted. Results were expressed in % ratio.

(c) Sperm viability was assessed using double fluorescent stain calcein-AM (C-AM; 1 mmol/L) and propidium iodide (PI; 0.75 mmol/L). Briefly, 100 μL of semen were mixed with 5 μL of C-AM and 1 μL of PI and incubated at 37 °C in the dark for 15 min. Sperm samples were evaluated using a fluorescence microscope (×400, AXIO Scope A1, Zeiss, Jena, Germany). Spermatozoa with intact plasma membrane fluoresce green, while the dead spermatozoa fluoresce red. In total, 200 spermatozoa per slide were estimated, and the results were expressed as percentage of live spermatozoa per sample.

(d) Sperm membrane functional status was assessed by hypoosmotic swelling test (HOST), which was applied as previously described [70] under slight modification. HOST solution was prepared with fructose (75 mmol/L) and sodium citrate (32 mmol/L), and the osmolality was adjusted to 150 mOsm using a cryoscopic osmometer (OSMOMAT^®^ 030, Gonotec, Berlin, Germany). Briefly, 100 μL of semen sample were mixed with 1 mL of HOST solution and incubated for 1 h at 37 °C. Thereafter, plasma membrane functional spermatozoa provided swollen tails. In total, 200 spermatozoa per slide were evaluated (×400, AXIO Scope A1, Zeiss, Jena, Germany). Results were expressed as percentage of spermatozoa with swollen tails.

(e) Sperm nuclear chromatin integrity was evaluated by the acridine orange test (AOT), which measures the susceptibility of sperm nuclear DNA to acid-induced denaturation in situ by quantifying the metachromatic shift of acridine orange fluorescence from green (native DNA) to red (denatured DNA). Acridine orange stains normal double-stranded DNA green and denatured single-stranded DNA red. All slides were examined under a fluorescence microscope (×1000, AXIO Scope A1, Zeiss, Jena, Germany) and 200 spermatozoa per slide were assessed in ten different optical areas for determination of percentage of spermatozoa with denatured DNA.

In order to clarify the type of DON and ZEN interaction on boar semen, we followed the approach of Grenier and Oswald [43]. Briefly the following categories of interactions were evaluated for each parameter tested in this study: synergism (type 1, 2, 3), additive interaction, less than additive interaction, and antagonism (type 1, 2). Detailed descriptions of the various categories can be retrieved from the aforementioned publication [43].

### 5.4. Statistical Analysis

In order to select the appropriate sample size *N* for the main trial experimental design, an a priori power analysis [71] was carried out, in which sample size *N* was computed as a function of the required power level (1-*β*), the pre-specified significance level (*α*), and the population effect size to be detected with probability (1-*β*). For the predefined power level (1-*β*) and the significance level (*α*) we had to make a choice in order to retain a reasonable balance between alpha and beta risk although there is no rule of thumb regarding the appropriate levels. Due to this fact, we followed recommendations [71] that claimed that the power and significance levels should be set at *β* = 0.80 and *α* = 0.05. Moreover, four-to-one weighting of beta-to-alpha risk serves as a good default that is reasonable in many settings [71]. Statistical software G*Power (version 3.1.9) was used for respective sample size calculations [72].

In order to investigate the effect of the factors’ treatment (i.e., mycotoxin effect) and time on the examined parameters, the same statistical approach was followed in both parts of the study. More specifically, the linear mixed effects (LME) modeling [73], a statistical methodology that is able to model both fixed and random effects, was used. Regarding the fixed effects (i.e., factors that may affect the mean values of the examined parameters), the design involves the investigation of the two main effects, which are (i) the main effect of factor treatment (i.e., mycotoxin effect (A)) (between factors) and (ii) the main effect of factor time (B) (within factors). In addition to the abovementioned two main effects, there is also a need to examine the interaction effect of treatment × time (A × B) (within-between factors), since the mycotoxin effect may depend on the time of the derived measurements. The optimal fixed component structure providing information regarding the main and interaction effects was defined through the protocol proposed by Zuur et al. [74]. Described briefly, a model examining all factors of interest (treatment and time) and their interaction (treatment × time) was fitted and tested against a second model after omitting the interaction term through the likelihood ratio (LR) test. In case of an insignificant interaction term, the selection is based on the principle of parsimony, which practically means that the model incorporating only the main effects was finally fitted on the data.

All statistical analyses were conducted using the statistical language R [75] and the function lmer from package lme4. In addition, the function step from package lmer Test [76] was used in order to perform backward elimination of all effects of the examined LME. The *p*-values for the fixed component of the model were calculated from *F* test based on Kenward–Roger approach in order to get approximate degrees of freedom [77]. In all tests a difference was considered as significant when *p*-value (significance) was less than 0.05. All tests conducted were two-tailed (non-directional) as the alternative hypothesis is that the measures tested are not equal.

## Figures and Tables

**Table 1:** Descriptive statistics of major computer-assisted semen analysis (CASA) measurements of the main trial (mean values ± standard deviation) at each observation time (0–4 h). Number of replicates = 10 in each test.

Treatments ^#^	0 h	1 h	2 h	3 h	4 h	*p* * (DMSO vs. Mycotoxin(s) Treatment)
**Immotile Spermatozoa (%)**						
DMSO	2.76 ± 1.72	4.01 ± 3.11	5.48 ± 3.08	8.67 ± 4.18)	10.70 ± 5.34	
DON	3.95 ± 2.43	6.11 ± 2.94	8.32 ± 3.84	12.09 ± 5.28	17.63 ± 10.90	0.001
ZEN	13.87 ± 10.98	21.95 ± 16.75	31.11 ± 19.02	32.37 ± 20.55	38.83 ± 18.69	<0.001
DON + ZEN	19.22 ± 9.55	19.17 ± 11.82	25.77 ± 8.16	34.13 ± 14.27	35.98 ± 15.51	<0.001
**Non-progressive Motile Spermatozoa (%)**						
DMSO	19.35 ± 3.82	22.37 ± 6.03	21.67 ± 4.25	20.60 ± 3.52	21.19 ± 4.55	
DON	19.88 ± 2.18	24.18 ± 6.13	24.79 ± 2.83	24.76 ± 4.10	25.33 ± 8.43	0.001
ZEN	34.02 ± 5.94	34.20 ± 11.17	33.81 ± 9.21	36.40 ± 11.52	33.21 ± 11.33	<0.001
DON + ZEN	41.31 ± 9.91	39.64 ± 12.04	44.07 ± 8.32	39.87 ± 10.90	38.50 ± 8.87	<0.001
**Progressive Motile Spermatozoa (%)**						
DMSO	77.88 ± 4.68	73.62 ± 7.13	72.85 ± 6.53	70.73 ± 6.12	68.11 ± 6.64	
DON	76.17 ± 3.53	69.71 ± 8.28	66.89 ± 5.70	63.14 ± 7.65	57.04 ± 16.61	0.056
ZEN	52.11 ± 14.24	43.85 ± 21.09	35.08 ± 18.95	31.24 ± 19.52	27.95 ± 17.81	<0.001
DON + ZEN	39.48 ± 18.70	41.19 ± 20.20	30.16 ± 11.62	26.00 ± 14.57	25.52 ± 16.43	<0.001
**Rapid (%)**						
DMSO	73.75 ^a^ ± 15.03	61.51 ^a^ ± 18.80	53.72 ^a^ ± 17.03	47.18 ^a^ ± 15.10	43.65 ^a^ ± 12.82	
DON	74.02 ^a^ ± 15.48	53.86 ^a^ ± 18.39	44.95 ^a^ ± 16.06	39.33 ^a^ ± 12.86	33.47 ^a^ ± 13.16	4 h: 0.063 **
ZEN	42.00 ^b^ ± 13.66	31.43 ^b^ ± 20.68	22.99 ^b^ ± 15.56	20.84 ^b^ ± 16.26	19.80 ^b^ ± 14.31	Each time point: <0.001 **
DON + ZEN	28.24 ^c^ ± 16.05	31.40 ^b^ ± 21.67	16.93 ^b^ ± 10.90	15.65 ^b^ ± 11.15	15.01 ^b^ ± 10.61	Each time point: <0.001 **
**Medium (%)**						
DMSO	17.36 ^a^ ± 9.5	24.35 ^a^ ± 9.86	27.91 ^ac^ ± 7.59	31.87 ^a^ ± 6.6	31.44 ^a^ ± 8.14	
DON	15.79 ^a^ ± 8.07	26.68 ^a^ ± 9.47	31.26 ^a^ ± 7.88	31.78 ^a^ ± 5.01	32.31 ^a^ ± 8.29	
ZEN	23.17 ^a^ ± 5.97	22.34 ^a^ ± 6.51	18.96 ^c^ ± 7.92	20.00 ^b^ ± 7.76	18.14 ^b^ ± 7.53	0 h: 0.08, 2 h: 0.008, 3 h, 4 h: <0.001 **
DON + ZEN	22.17 ^a^ ± 6.96	21.02 ^a^ ± 4.85	21.89 ^bc^ ± 6.24	19.11 ^b^ ± 6.87	21.37 ^b^ ± 8.13	2 h: 0.07, 3 h & 4 h: <0.001 **
**Slow (%)**						
DMSO	6.12 ^a^ ± 4.89	10.14 ^a^ ± 7.40	12.88 ^a^ ± 7.43	12.27 ^a^ ± 5.19	12.21 ^a^ ± 3.07	
DON	6.24 ^a^ ± 5.91	13.34 ^a^ ± 8.03	15.47 ^a^ ± 6.50	16.79 ^a^ ± 5.82	16.59 ^ab^ ± 4.12	1 h: 0.062 **
ZEN	20.95 ^b^ ± 7.15	24.28 ^b^ ± 9.38	26.94 ^b^ ± 9.64	26.79 ^b^ ± 10.37	23.22 ^bc^ ± 8.20	0 h, 1 h, 2 h, 3 h: <0.001 ** 4 h: 0.006
DON + ZEN	30.37 ^b^ ± 11.70	28.41 ^b^ ± 11.62	35.40 ^b^ ± 7.18	31.11 ^b^ ± 8.79	27.63 ^c^ ± 6.77	Each time point: <0.001 **
**VCL (Curvilinear Velocity; μm/s)**						
DMSO	74.36 ^a^ ± 19.10	61.90 ^a^ ± 15.31	59.21 ^a^ ± 21.78	51.08 ^a^ ± 9.86	48.12 ^a^ ± 5.88	
DON	74.01 ^a^ ± 16.57	57.27 ^a^ ± 13.69	51.44 ^a^ ± 10.90	45.22 ^a^ ± 8.12	41.88 ^ab^ ± 7.99	2 h: 0.098 **
ZEN	50.09 ^b^ ± 10.69	43.02 ^b^ ± 13.77	37.74 ^b^ ± 10.65	35.72 ^b^ ± 12.42	35.55 ^bc^ ± 9.96	0 h, 1 h, 2 h: <0.001, 3 h: 0.001, 4 h: 0.008 **
DON + ZEN	39.96 ^c^ ± 11.87	44.38 ^b^ ± 18.49	33.92 ^b^ ± 9.59	32.63 ^b^ ± 9.19	32.43 ^c^ ± 7.46	0 h, 1 h, 2 h, 3 h: <0.001 ** 4 h: 0.001
**VSL (Straight-line Velocity; μm/s)**						
DMSO	32.46 ± 3.55	32.46 ± 2.60	33.58 ± 5.71	30.83 ± 3.81	28.81 ± 5.08	
DON	32.71 ± 2.16	30.57 ± 3.31	29.35 ± 2.18	27.18 ± 5.29	24.58 ± 7.46	
ZEN	18.57 ± 4.82	19.05 ± 8.91	15.74 ± 7.74	14.46 ± 10.03	12.33 ± 8.90	<0.001
DON + ZEN	14.43 ± 7.52	19.12 ± 12.25	13.60 ± 6.22	12.74 ± 9.04	10.47 ± 6.45	<0.001
**VAP (Average Path Velocity; μm/s)**						
DMSO	45.19 ± 5.96	42.18 ± 4.42	42.59 ± 9.02	38.36 ± 4.73	36.21 ± 5.14	
DON	45.78 ± 4.66	38.91 ± 4.03	37.43 ± 3.34	33.81 ± 6.08	30.72 ± 7.81	0.089
ZEN	28.19 ± 6.32	26.33 ± 10.39	22.06 ± 8.19	20.60 ± 10.84	18.20 ± 9.10	<0.001
DON + ZEN	22.07 ± 9.57	26.90 ± 14.05	19.70 ± 6.74	18.53 ± 9.47	16.31 ± 6.85	<0.001
**LIN (Linearity; %)**						
DMSO	45.62 ^a^ ± 9.8	54.94 ^a^ ± 12.1	60.16 ^a^ ± 12.73	62.08 ^a^ ± 12.03	60.21 ^a^ ± 10.48	
DON	46.15 ^a^ ± 10.14	56.25 ^a^ ± 14.69	59.59 ^a^ ± 14.06	61.21 ^a^ ± 12.35	59.49 ^a^ ± 17.55	
ZEN	37.39 ^b^ ± 7.47	43.35 ^b^ ± 14.24	41.06 ^b^ ± 15.28	38.43 ^b^ ± 20.01	33.68 ^b^ ± 18.74	0 h: 0.028, 1 h: 0.002, 2 h,3 h, 4 h: <0.001 **
DON + ZEN	34.16 ^b^ ± 10.36	41.30 ^b^ ± 14.47	40.52 ^b^ ± 17.56	37.21 ^b^ ± 19.45	31.98 ^b^ ± 17.6	0 h: 0.002, 1 h,2 h,3 h, 4 h: <0.001 **
**STR (Straightness; %)**						
DMSO	72.08 ^a^ ± 4.07	77.20 ^a^ ± 3.64	79.37 ^a^ ± 4.25	80.45 ^a^ ± 3.62	79.16 ^a^ ± 3.79	
DON	71.76 ^a^ ± 4.06	78.64 ^a^ ± 4.72	78.66 ^a^ ± 5.06	80.29 ^a^ ± 3.92	78.85 ^a^ ± 7.96	
ZEN	65.50 ^b^ ± 4.16	70.09 ^b^ ± 8.27	68.12 ^b^ ± 12.13	63.74 ^b^ ± 16.32	62.87 ^b^ ± 12.78	0 h: 0.033, 1 h: 0.022, 3 h, 4 h: <0.001 **
DON + ZEN	62.61 ^b^ ± 7.70	67.64 ^b^ ± 10.08	66.23 ^b^ ± 12.06	63.12 ^b^ ± 14.46	59.75 ^b^ ± 14.63	0 h & 1 h: 0.002, 2 h,3 h, 4 h: <0.001 **
**Wobble (WOB; %)**						
DMSO	62.92 ^a^ ± 10.79	70.66 ^a^ ± 12.26	75.26 ^a^ ± 12.23	76.72 ^a^ ± 11.6	75.68 ^a^ ± 10.18	
DON	63.89 ^a^ ± 10.78	70.79 ^a^ ± 14.43	75.06 ^a^ ± 13.11	75.75 ^a^ ± 12.13	74.03 ^a^ ± 16.52	
ZEN	56.70 ^a^ ± 7.84	60.55 ^b^ ± 13.47	58.34 ^b^ ± 13.92	56.44 ^b^ ± 18.55	50.45 ^b^ ± 17.86	0 h: 0.057, 1 h: 0.002, 2 h,3 h, 4 h: <0.001 **
DON + ZEN	53.49 ^b^ ± 10.45	59.39 ^b^ ± 13.52	58.69 ^b^ ± 16.25	55.43 ^b^ ± 17.88	50.09 ^b^ ± 16.84	0 h: 0.004, 1 h: 0.001, 2 h,3 h, 4 h: <0.001 **
**ALH (Amplitude of Lateral Head Displacement; μm)**						
DMSO	1.89 ^a^ ± 0.26	1.64 ^a^ ± 0.2	1.60 ^a^ ± 0.4	1.41 ^a^ ± 0.14	1.39 ^a^ ± 0.07	
DON	1.90 ^a^ ± 0.24	1.58 ^a^ ± 0.2	1.48 ^a^ ± 0.17	1.32 ^a^ ± 0.17	1.26 ^a^ ± 0.18	
ZEN	1.74 ^a^ ± 0.12	1.51 ^a^ ± 0.19	1.51 ^a^ ± 0.14	1.40 ^a^ ± 0.09	1.43 ^a^ ± 0.19	0 h: 0.074 **
DON + ZEN	1.59 ^b^ ± 0.24	1.57 ^a^ ± 0.18	1.42 ^b^ ± 0.17	1.41 ^a^ ± 0.13	1.36 ^a^ ± 0.17	0 h: <0.001, 2 h: 0.033 **
**BCF (Beat/Cross Frequency; Hz)**						
DMSO	13.13 ± 2.81	11.80 ± 2.41	10.60 ± 2.25	10.50 ± 1.79	10.32 ± 1.83	
DON	12.70 ± 2.03	11.14 ± 2.37	10.05 ± 1.87	9.73 ± 1.47	9.36 ± 1.72	
ZEN	9.72 ± 1.58	9.04 ± 2.60	9.17 ± 2.63	7.95 ± 3.51	7.64 ± 2.19	0.001
DON + ZEN	7.93 ± 2.08	8.3 ± 11.97	8.00 ± 2.97	7.13 ± 2.39	6.05 ± 2.53	<0.001
**Hyperactive (%)**						
DMSO	0.031 ^a^ ± 0.02	0.022 ^a^ ± 0.017	0.022 ^a^ ± 0.033	0.007 ^a^ ± 0.008	0.005 ^a^ ± 0.005	
DON	0.031 ^a^ ± 0.019	0.018 ^ab^ ± 0.014	0.010 ^b^ ± 0.010	0.006 ^a^ ± 0.006	0.004 ^a^ ± 0.004	2 h: 0.024 **
ZEN	0.010 ^b^ ± 0.005	0.011 ^b^ ± 0.013	0.005 ^b^ ± 0.007	0.003 ^a^ ± 0.004	0.001 ^a^ ± 0.001	0 h: 0.000, 1 h: 0.036, 2 h: 0.001 **
DON + ZEN	0.009 ^b^ ± 0.011	0.016 ^ab^ ± 0.02	0.004 ^b^ ± 0.007	0.002 ^a^± 0.003	0.001 ^a^ ± 0.001	0 h: 0.000, 2 h: 0.001 **

^a,b,c^ Mean values with different superscripts in the same column differ significantly (*p* < 0.05). * When *p*-values are reported without superscripts in relevant parameter mean values, they refer to statistically significant main effect (*p* < 0.05) or trend towards statistical significance (0.05 < *p* < 0.1) of treatment (mycotoxin) on the response variable, without significant interaction term treatment × time, thus differences refer to the total observation period. ** When *p*-values are reported and superscripts (^a,b,c^) are placed in relevant parameter mean values, they refer to statistically significant main effect (*p* < 0.05) or trend towards statistical significance (0.05 < *p* < 0.1) of treatment (mycotoxin) on the response variable, with a statistically significant interaction term treatment × time on the response variable present, thus differences refer to specific time points (0–4 h of investigation). # Treatments: dimethyl sulfoxide (DMSO) = 0.7% (*v*/*v*); deoxynivalenol (DON) = 50.6 μM; zearalenone (ZEN) = 62.8 μM; DON + ZEN = 50.6 μM DON + 62.8 μM ZEN.

**Table 2:** Descriptive statistics of semen traits (mean values ± standard deviation) at each observation time (0–4 h of incubation). Number of replicates = 10 in each test.

Morphology (% Spermatozoa with Head Abnormalities)						
Treatments #	0 h	1 h	2 h	3 h	4 h	*p* * (DMSO vs. Mycotoxin(s) Treatment)
DMSO	9.70 ± 4.69	13.30 ± 5.76	16.10 ± 5.57	21.10 ± 6.57	22.40 ± 7.63	
DON	11.00 ± 6.43	13.10 ± 5.65	20.80 ± 7.81	23.85 ± 9.46	24.15 ± 10.23	
ZEN	25.60 ± 17.48	32.00 ± 19.58	35.30 ± 21.88	36.60 ± 22.19	40.60 ± 22.16	0.002
DON + ZEN	31.20 ± 21.81	35.10 ± 18.82	43.40 ± 20.44	43.80 ± 23.99	46.70 ± 22.88	<0.001
**Viability (% Live Spermatozoa)**						
DMSO	81.70 ^a^ ± 7.18	78.30 ^a^ ± 6.63	76.00 ^a^ ± 9.12	71.00 ^a^ ± 16.58	67.80 ^a^ ± 18.70	
DON	80.30 ^a^ ± 8.45	76.80 ^a^ ± 8.30	71.50 ^a^ ± 10.87	65.90 ^a^ ± 12.15	59.70 ^b^ ± 15.38	4 h: 0.041 **
ZEN	66.60 ^b^ ± 11.34	50.20 ^b^ ± 12.97	37.80 ^b^ ± 13.22	34.60 ^b^ ± 12.77	30.80 ^c^ ± 12.21	Each time point: <0.001 **
DON + ZEN	54.10 ^c^ ± 14.69	37.00 ^c^ ± 9.67	27.70 ^c^ ± 9.38	24.10 ^c^ ± 11.13	21.80 ^d^ ± 10.26	Each time point: <0.001 **
**Hypoosmotic Swelling Test (HOST, % Spermatozoa with Swollen Tails)**						
	0 h	1 h	4 h	*p* * (DMSO vs. Mycotoxin(s) Treatment)		
DMSO	22.90 ± 9.81	14.90 ± 4.98	8.30 ± 2.95			
DON	19.80 ± 7.38	12.25 ±2.49	8.05 ±3.00	0.047		
ZEN	14.40 ± 5.62	8.50 ± 3.41	4.40 ± 3.81	<0.001		
DON + ZEN	12.60 ± 4.95	8.00 ± 4.55	3.60 ± 3.63	<0.001		

^a,b,c,d^ Mean values with different superscripts in the same column differ significantly (*p* < 0.05). * When *p*-values are reported without superscripts in relevant parameter mean values, they refer to statistically significant main effect of treatment (mycotoxin) on the response variable, without significant interaction term treatment × time, thus differences refer to the total observation period. ** When *p*-values are reported and superscripts (^a,b,c,d^) are placed in relevant parameter mean values, a statistically significant interaction term treatment × time on the response variable was present, thus differences refer to specific time points (0–4 h of investigation). ^#^ Treatments: DMSO = 0.7% (*v*/*v*); DON = 50.6 μM; ZEN = 62.8 μM; DON + ZEN = 50.6 μM DON + 62.8 μM ZEN.

**Table 3:** Combined effects and interactions between DON and ZEN on boar semen exposed in vitro (based on [43]).

**CASA Parameters**	**Mean Values Comparisons and Alterations ^#^**	**Combined Effect and Type of Interaction (0–4 h of Incubation)**
Immotile	DON + ZEN vs. ZEN: *p* > 0.05 and DON + ZEN vs. DON: *p* < 0.05 and DON vs. ZEN: *p* < 0.05. No treatment × time interaction.	Less than additive
Non progressive	DON + ZEN vs. ZEN: *p* > 0.05 and DON + ZEN vs. DON: *p* < 0.05 and DON vs. ZEN: *p* < 0.05. No treatment × time interaction.	Less than additive
Progressive motile	DON + ZEN vs. ZEN: *p* > 0.05 and DON + ZEN vs. DON: *p* < 0.05 and DON vs. ZEN: *p* < 0.05. No treatment × time interaction.	Less than additive
Rapid	Treatment × time interaction was observed. All time points: DON + ZEN vs. DON: *p* < 0.05 and DON vs. ZEN: *p* < 0.05. DON + ZEN vs. ZEN: 0 h: *p* < 0.05; 1–4 h: *p* > 0.05.	0 h: Potentiation (synergism type 1) effect (DON + ZEN vs. ZEN: *p* < 0.05) 1–4 h: Less than additive
Medium	Treatment × time interaction was observed. DON vs. ZEN: 0 h and 2–4 h *p* < 0.05; 1 h: *p* > 0.05. All time points DON + ZEN vs. ZEN: *p* > 0.05. DON + ZEN vs. DON: 0 h and 1 h: *p* > 0.05; 2–4 h: *p* < 0.05.	0 h and 1 h: No interaction 2–4 h: Less than additive (type II)
Slow	Treatment × time interaction was observed. All time points DON + ZEN vs. ZEN: *p* > 0.05 and DON + ZEN vs. DON: *p* < 0.05. DON vs. ZEN: 0–3 h: *p* < 0.05; 4 h: *p* > 0.05.	Less than additive
VCL	Treatment × time interaction was observed. DON + ZEN vs. ZEN: 0 h: *p* < 0.05; 1–4 h: *p* > 0.05. All time points: DON + ZEN vs DON: *p* < 0.05. DON vs ZEN: 0–3 h: *p* < 0.05; 4 h: *p* > 0.05.	0 h: Potentiating interaction (synergism type 1) 1–4 h: Less than additive
VSL	Similar to parameter “Immotile”	Less than additive
VAP	Similar to parameter “Immotile”	Less than additive
Linearity	Treatment × time interaction was observed. All time points: DON + ZEN vs. ZEN: *p* > 0.05 and DON + ZEN vs. DON: *p* < 0.05 and DON vs. ZEN: *p* < 0.05.	Less than additive
Straightness	Treatment × time interaction was observed. All time points: DON + ZEN vs. ZEN: *p* > 0.05 and DON + ZEN vs. DON: *p* < 0.05 and DON vs. ZEN: *p* < 0.05.	Less than additive
ALH	Treatment × time interaction was observed. 0 h and 2 h: DON + ZEN vs. DON: *p* < 0.05; DON + ZEN vs. ZEN: *p* > 0.05; DON vs. ZEN: *p* > 0.05. 1 h, 3 h, 4 h: No significant effect of all treatments on the parameter.	0 h and 2 h: Less than additive 1 h, 3 h, and 4 h: No interaction
BCF*	Similar to parameter “Immotile”	Less than additive
Wobble	Treatment × time interaction was observed. All time points: DON + ZEN vs. ZEN: *p* > 0.05 and DON + ZEN vs. DON: *p* < 0.05 and DON vs. ZEN: *p* < 0.05.	Less than additive
Hyperactive	Treatment × time interaction was observed. All time points: DON + ZEN vs. ZEN: *p* > 0.05; DON + ZEN vs. DON: 0 h: *p* < 0.05; 1–4 h: *p* > 0.05. DON vs. ZEN: 0 h: *p* < 0.05; 1–2 h: *p* > 0.05; 3–4 h: No significant effect of all treatments on the parameter.	0 h and 2 h: Less than additive 1 h, 3 h, and 4 h: No interaction
**Morphology, Viability, HOST, and DNA Integrity Tests**		
Morphology/Head incidents	DON + ZEN vs. ZEN: *p* > 0.05 and DON + ZEN vs. DON: *p* < 0.05 and DON vs. ZEN: *p* < 0.05. No treatment × time interaction.	Less than additive
Viability/Live spermatozoa	Treatment × time interaction was observed. All time points: DON + ZEN vs. ZEN: *p* < 0.05 and DON + ZEN vs. DON: *p* < 0.05 and DON vs. ZEN: *p* < 0.05.	Synergistic effect (type 1)
Sperm membrane functional status/HOST	All time points: DON + ZEN vs. ZEN: *p* > 0.05 and DON + ZEN vs. DON: *p* < 0.05 and DON vs. ZEN: *p* < 0.05. No treatment × time interaction.	Less than additive
Sperm nuclear chromatin integrity	–	No interaction

^#^ Treatments: DMSO = 0.7% (*v*/*v*); DON = 50.6 μM; ZEN = 62.8 μM; DON + ZEN = 50.6 μM DON + 62.8 μM ZEN; * BCF: beat/cross frequency; HOST: hypoosmotic swelling test.

## Supplementary Materials

The following are available online at https://www.mdpi.com/2072-6651/12/8/495/s1, Figure S1: Distribution of progressive motility values for control and DMSO concentrations per hour (0–4 h) of evaluation. DMSO concentrations (*v*/*v*) used were DMSO 0.7%, DMSO 0.8%, DMSO 0.9%, DMSO 1.2%, DMSO 3%, DMSO 5%; Figure S2: Pretrial tests’ distribution of progressive motility (Pro) mean values of DON-treated extended boar semen per hour of investigation (0–4 h). DMSO concentration (*v*/*v*) used was 0.7% in all groups, except for the control. Presented DON treatments were: 16.9 μM DON, 33.8 μM DON, 50.6 μM DON, 67.5 μM DON; Figure S3: Pretrial tests distribution of progressive motility (Pro) mean values of ZEN-treated extended boar semen per hour of investigation (0–4 h). DMSO concentration (*v*/*v*) used was 0.7% in all groups, except for the control. Presented ZEN treatments were: 31.4 μM ZEN, 47.1 μM ZEN, 62.8 μM ZEN, 94.2 μM ZEN, 125.6 μM ZEN; Figure S4: Morphology evaluated by SpermBlue staining method (×400). Spermatozoon (ZEN group, 4 h) without tail (marked with *), swelling acrosome (marked with an arrow); Figure S5: Double fluorescent stain calcein-AM and propidium iodide (×200). Spermatozoa with intact plasma membrane fluoresce green (arrow), whereas the dead sperms fluoresce red (star sign) (ZEN group, 4 h); Figure S6: Hypo-osmotic swelling test (HOST) (×200), plasma membrane functional spermatozoa provide swollen-coiled tails (marked with an arrow) (ZEN group, 4 h); Figure S7: Acridine orange (AO) staining (×100). Normal spermatozoa with compact chromatin structure fluoresce green (arrow), whilst damaged spermatozoa with de-compacted chromatin (single-stranded DNA) fluoresce red (star sign) (DON + ZEN group, 4 h); Figure S8: Acridine orange (AO) staining (×200). Semen sample with normal spermatozoa with compact chromatin structure which fluoresce green (DMSO group, 0 h).
