# Supplementary material for: Individual and Combined In Vitro Effects of Deoxynivalenol and Zearalenone on Boar Semen
Source: Toxins (Basel). 2020 Aug 1;12(8):495. doi: 10.3390/toxins12080495 (PMC7472223; doi:10.3390/toxins12080495)
Supplement: Supplementary file 1 [file toxins-12-00495-s001.pdf]

# Supplementary Materials: Individual and Combined In Vitro Effects of Deoxynivalenol and Zearalenone on Boar Semen

**Panagiotis D. Tassis, Ioannis A. Tsakmakidis, Veronika Nagl, Nicole Reisinger, Eleni Tzika, Christiane Gruber-Dorninger, Ilias Michos, Nikolaos Mittas, Athina Basioura and Dian Schatzmayr**

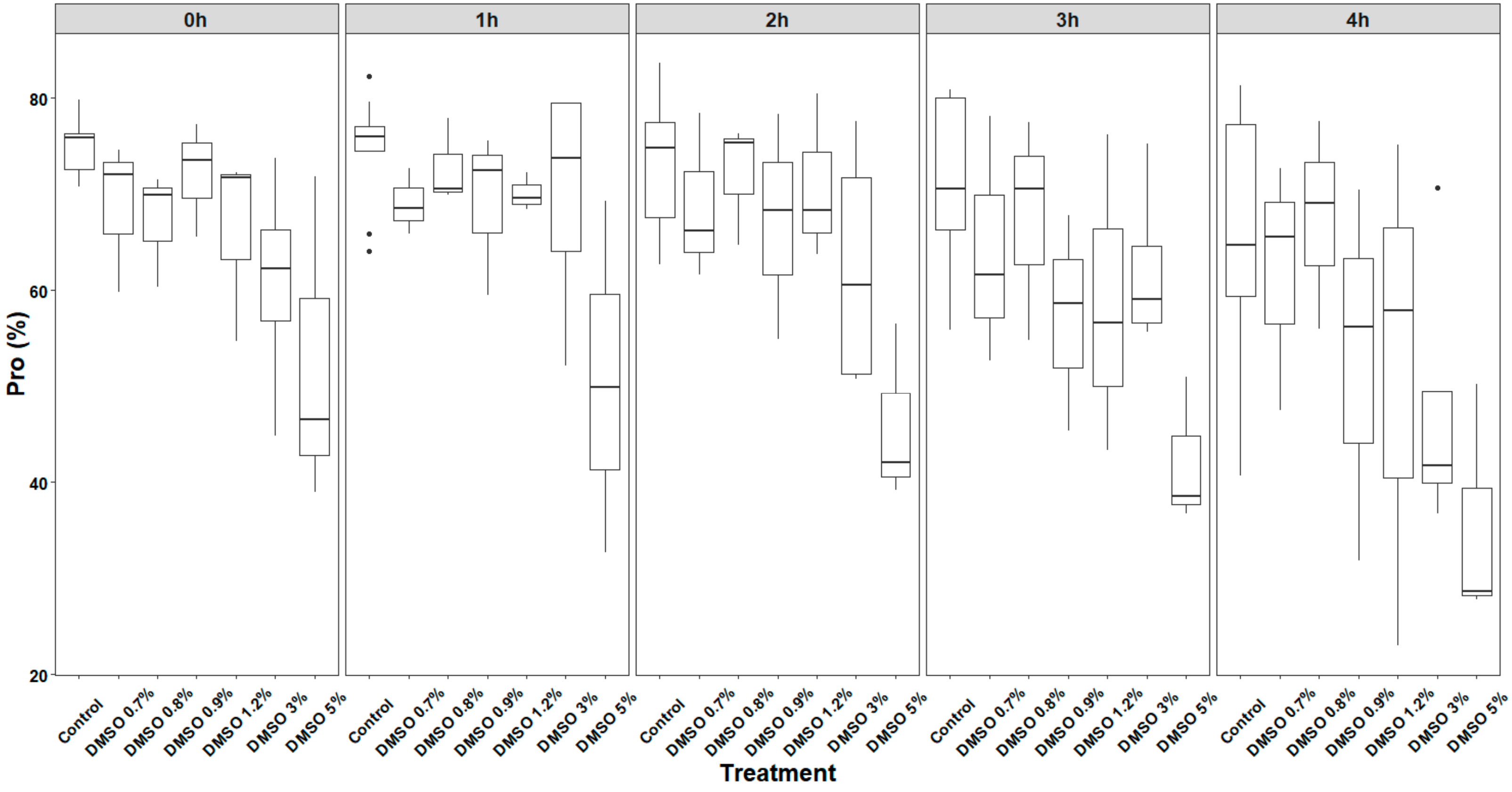


**Figure S1.** Distribution of progressive motility values for control and DMSO concentrations per hour (0–4 h) of evaluation (*n* = 5). DMSO concentrations (v/v) used were 0.7%, 0.8%, 0.9%, 1.2%, 3%, and 5%.

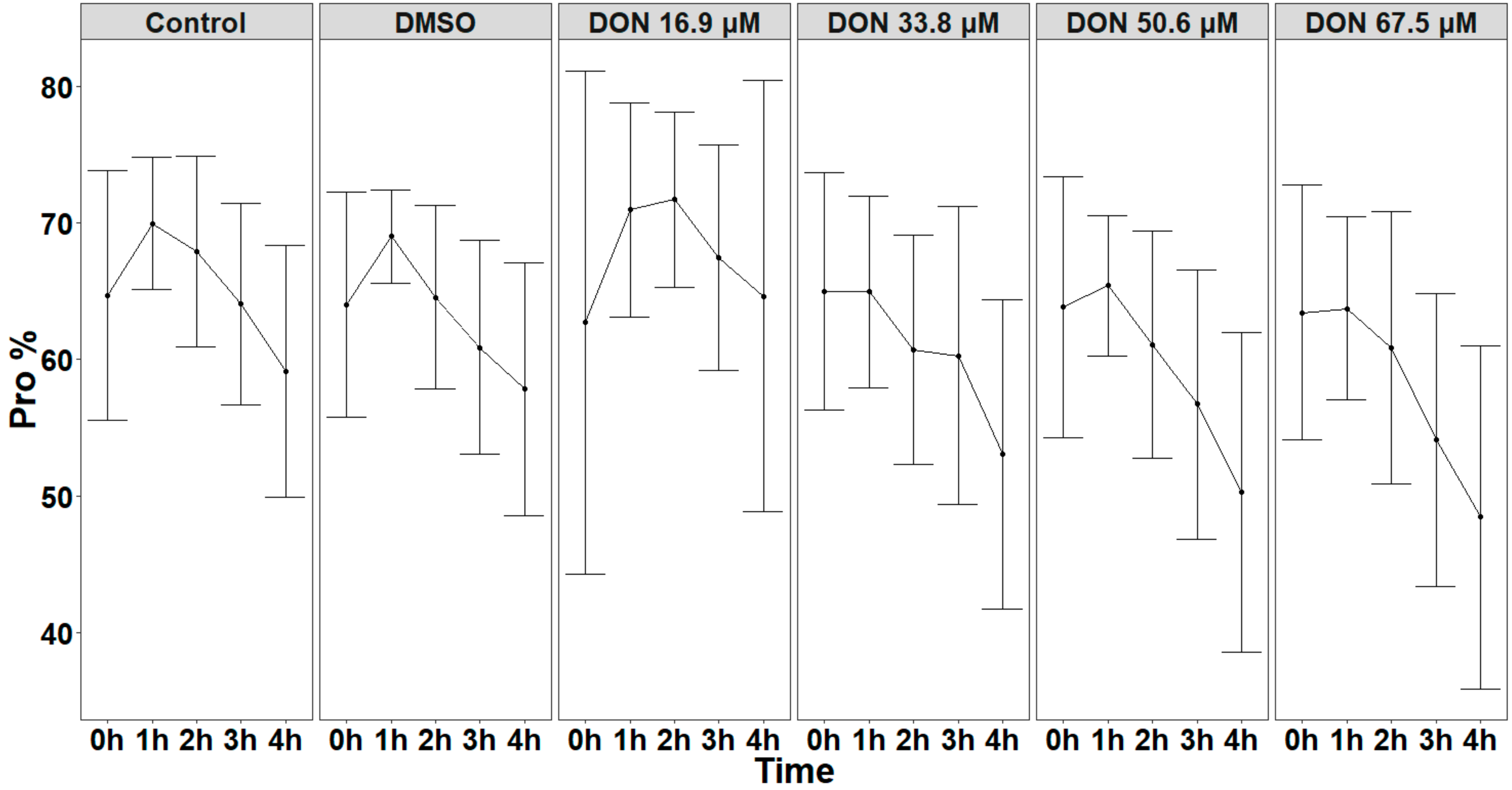


**Figure S2.** Tests' distribution of progressive motility (Pro) mean values of DON-treated extended boar semen per hour of investigation (0–4 h). DMSO concentration (v/v) used was 0.7% in all groups, except for the control (*n* = 5). Presented DON treatments were: 16.9 μM, 33.8 μM, 50.6 μM, and 67.5 μM.

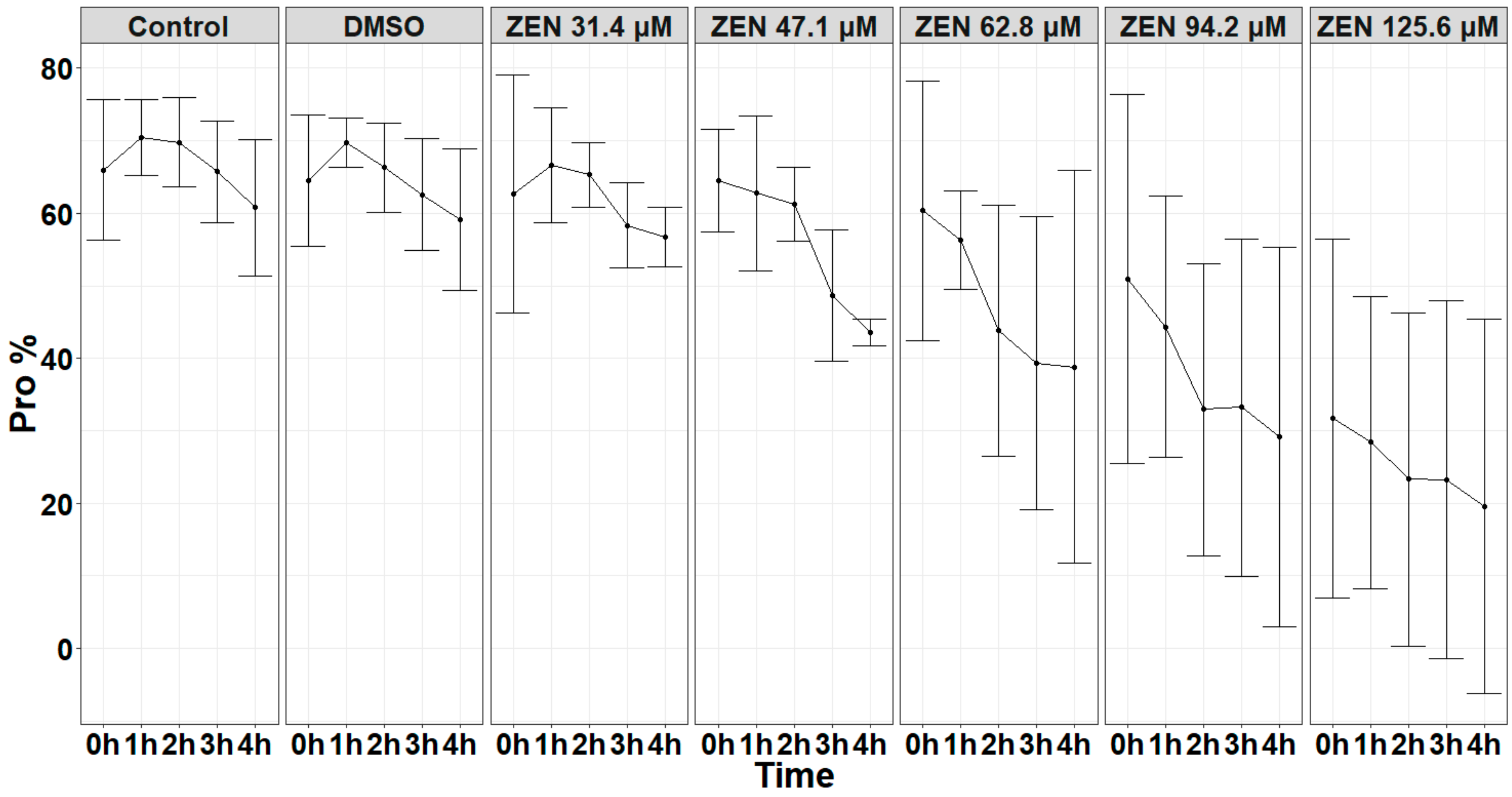


**Figure S3.** Pretrial tests' distribution of progressive motility (Pro) mean values of ZEN-treated extended boar semen per hour of investigation (0-4 h). DMSO concentration (v/v) used was 0.7% in all groups, except for the control (*n* = 5). Presented ZEN treatments are: 31.4 µM, 47.1 µM, 62.8 µM, 94.2 µM, and 125.6 µM.

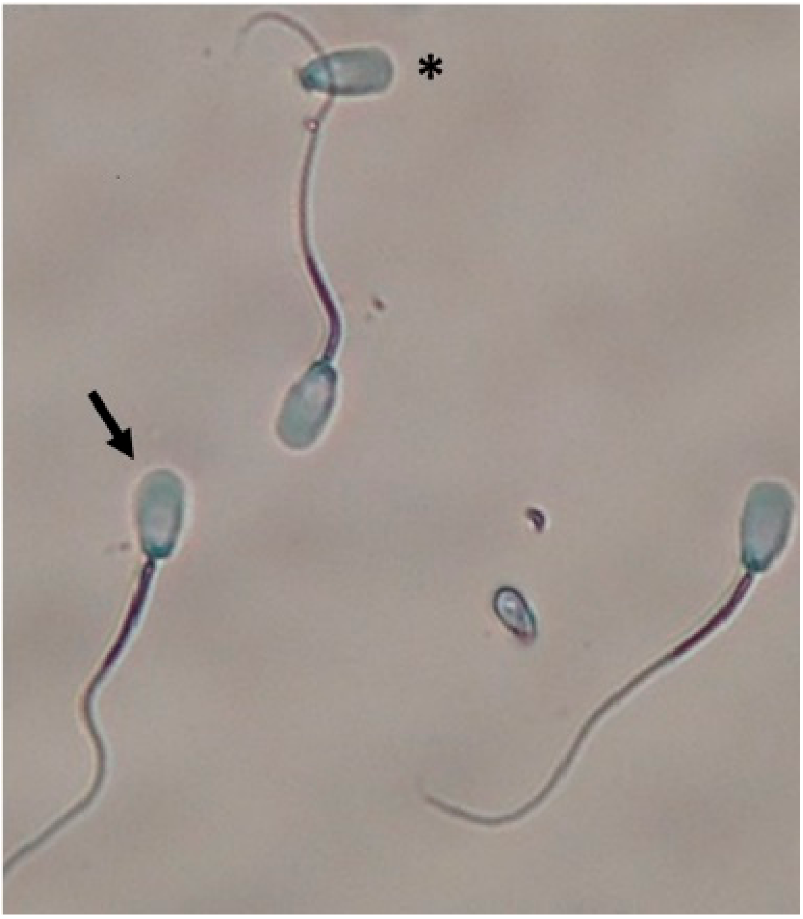


**Figure S4.** Morphology evaluated by SpermBlue staining method (×400). Spermatozoon (ZEN group, 4 h) without tail (marked with *), swelling acrosome (marked with an arrow).

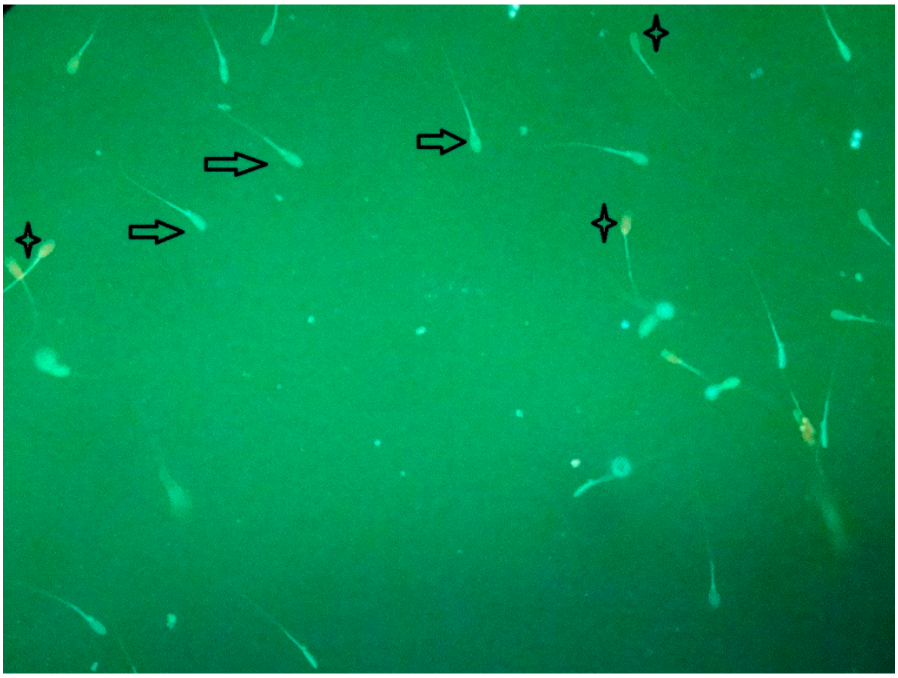

**Figure S5.** Double fluorescent stain calcein-AM and propidium iodide (×200). Spermatozoa with intact plasma membrane fluoresce green (arrow), whereas the dead sperms fluoresce red (star sign) (ZEN group, 4 h).

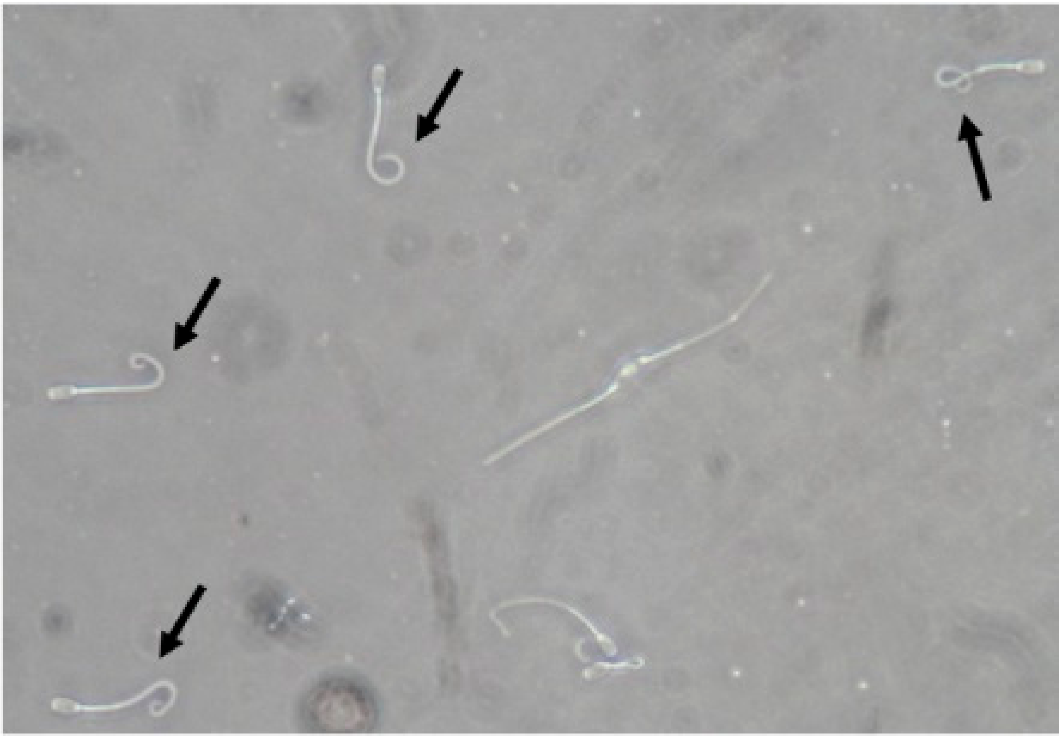

**Figure S6.** Hypo-osmotic swelling test (HOST) (×200), plasma membrane functional spermatozoa provide swollen-coiled tails (marked with an arrow) (ZEN group, 4 h).

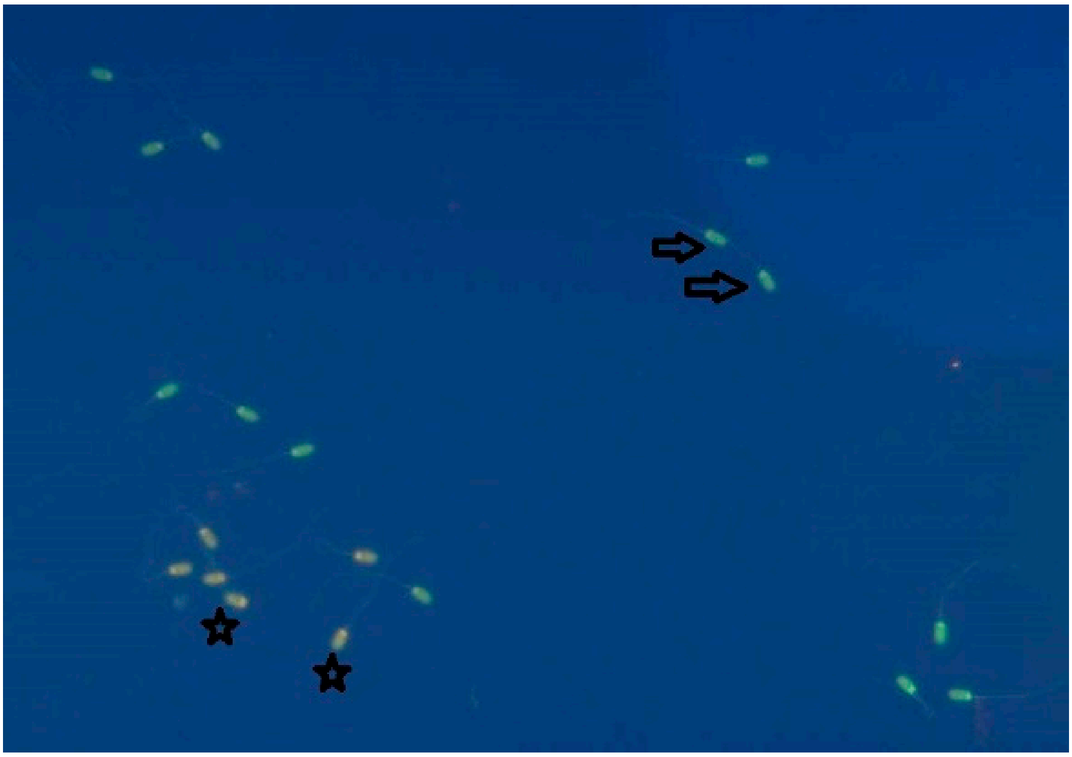

**Figure S7.** Acridine orange (AO) staining (×100). Normal spermatozoa with compact chromatin structure fluoresce green (arrow), whilst damaged spermatozoa with de-compacted chromatin (single-stranded DNA) fluoresce red (star sign) (DON + ZEN group, 4 h).

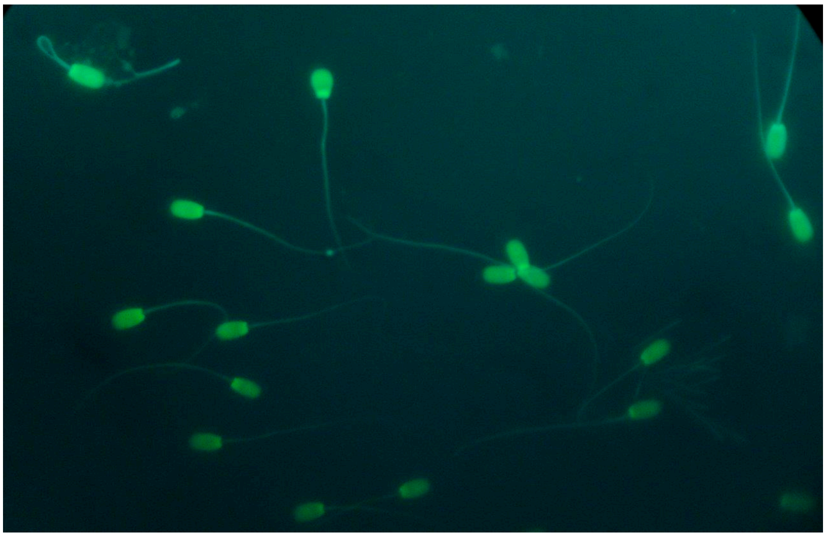

**Figure S8.** Acridine orange (AO) staining (×200). Semen sample with normal spermatozoa with compact chromatin structure fluoresce green (DMSO group, 0 h).
